# Time-Restricted Feeding Attenuates Adipose Tissue Inflammation and Fibrosis in Mice Under Chronic Light Exposure

**DOI:** 10.3390/ijms252111524

**Published:** 2024-10-26

**Authors:** Jiyeon Nah, Narae Yun, Hyunjin Yoo, Surin Park, Munkyong Pae

**Affiliations:** Department of Food and Nutrition, Chungbuk National University, Chungdae-ro 1, Seowon-gu, Cheongju 28644, Republic of Korea; nnjj0000@naver.com (J.N.); skfo1999@naver.com (N.Y.); yuhj610@naver.com (H.Y.); srhc2001@naver.com (S.P.)

**Keywords:** adipose tissue inflammation, chronic light, fibrosis, high-fat diet, time-restricted feeding

## Abstract

Time-restricted feeding (TRF) has emerged as a promising dietary approach for improving metabolic parameters associated with obesity. However, it remains largely unclear whether TRF offers benefits for obesity related to exposure to light at night. This study examined whether lean and obese mice under chronic light exposure could benefit from TRF intervention. Six-week-old C57BL/6 male mice were fed either a low-fat diet or a high-fat diet under a 12 h light/12 h dark cycle for 6 weeks. They were then divided into three subgroups: control light, chronic 24 h light, and chronic light with a daily 10 h TRF. Chronic light exposure led to increased weight gain and higher expression of inflammatory and fibrotic markers in the adipose tissue of both lean and obese mice. It also increased hepatic triglyceride content in mice, regardless of their weight status. TRF protected both lean and obese mice from weight gain, normalized inflammatory and fibrotic gene expression, and reduced adipose tissue collagen and liver triglyceride accumulation caused by light exposure alone or in combination with obesity. These results suggest that TRF could have clinical implications for preventing obesity associated with night shift work, regardless of current weight status.

## 1. Introduction

Excessive calorie intake and insufficient physical activity are the main factors responsible for obesity and its comorbidities, including type II diabetes and nonalcoholic fatty liver disease. However, environmental factors, such as light exposure at night, have also been implicated in the pathogenesis of obesity [1]. In recent decades, the global rise in obesity prevalence has coincided with increased exposure to light at night [2,3]. The availability of artificial light has substantially changed the light environment, especially during night hours, enabling non-stop economic activities. A recent study revealed a significant association between exposure to light at night while sleeping and the prevalence and incidence of obesity in a cohort of over 40,000 women [4]. In a cross-sectional study, night shift workers had shorter sleep durations and were almost three times more likely to have abdominal obesity, independent of age and gender, compared to day shift workers [5]. Rodent studies have shown that chronic light exposure at night increases susceptibility to weight gain and/or fat accumulation in lean mice [3,6,7], while exaggerating adipose tissue inflammation in a diet-induced obesity model of mice [8]. Although the mechanisms behind night-time light exposure and obesity remain largely elusive, accumulating evidence suggests that disrupted circadian (~24 h) rhythms, such as erratic eating patterns, play a crucial role in mediating metabolic dysfunction [3,8]. 

Time-restricted feeding (TRF) is a dietary regimen in which all caloric intake is restricted to a consistent interval, without an overt attempt to change the nutritional quantity and quality [9,10]. TRF provides protection against the development of, or preexisting, obesity, hepatic steatosis, and inflammation in rodents associated with a high-fat diet (HFD) [11,12,13]. While mice lacking core circadian clock genes tend to snack continuously on a HFD throughout the day and night, TRF effectively restores feeding–fasting rhythms and liver function in these mice [14]. We also demonstrated that TRF (10 h feed, 14 h fast cycles) can protect against glucose intolerance and preserve immune cell homeostasis, which were disturbed in diet-induced obese mice [15,16]. Despite the recognized beneficial effects of TRF, it remains largely unknown whether TRF provides metabolic protection in both lean and obese mice when challenged with night-time light exposure. 

In this study, we investigated whether chronic light exposure alone, or in combination with TRF, impacts adipose tissue dysregulation, including inflammation, fibrosis, and lipid metabolism, in both lean and obese mice. Since dysregulation of lipid metabolism in adipose tissue can lead to ectopic lipid storage in non-adipose tissue, we also examined hepatic steatosis and fibrosis, as determined by triglyceride content and gene expression related to inflammation and fibrosis. We found that TRF can be an effective approach for preventing weight gain associated with light at night in both lean and obese mice. Additionally, TRF attenuates adipose tissue inflammation and fibrosis, as well as hepatic steatosis induced by chronic light alone or in combination with a HFD.

## 2. Results

### 2.1. Effects of Chronic Light Exposure and TRF on Body Weight and Tissue Mass

**Figure 1:**
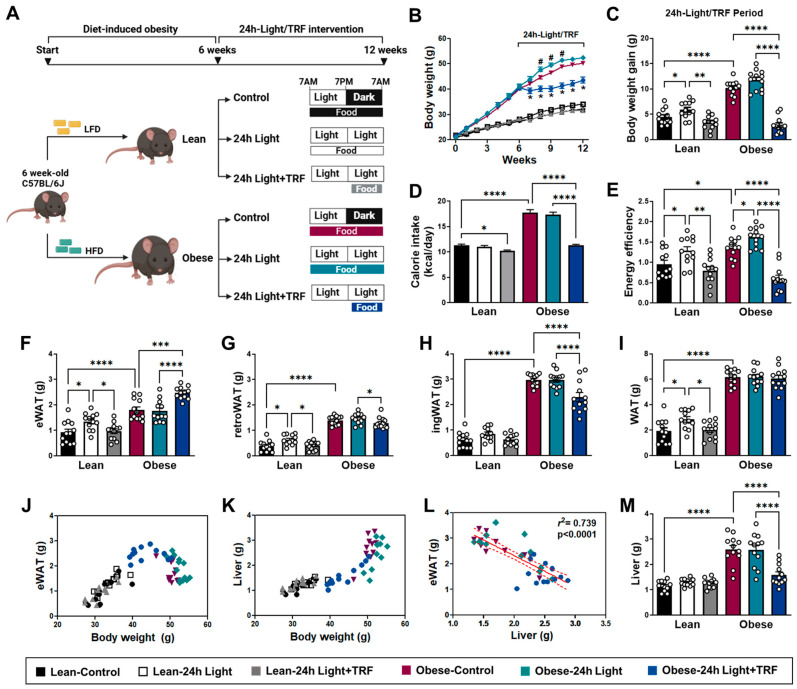
Effects of chronic light exposure and time-restricted feeding (TRF) on body weight and tissue mass. (**A**) Overview of the study design. Six-week-old C57BL/6J male mice were fed either a low-fat diet (LFD) or a high-fat diet (HFD) ad libitum under a control light condition (12 h light/dark cycle). After 6 weeks, the LFD-fed lean or HFD-fed obese mice were exposed for another 6 weeks to either a control light condition (12 h light/dark cycle), chronic 24 h light exposure, or chronic 24 h light exposure with a daily 10 h TRF intervention. (**B**) Body weight during the entire period. # *p* < 0.05 for Obese-24 h Light (vs. Obese-control); * *p* < 0.05 for Obese-24 h Light+TRF (vs. Obese-24 h Light). (**C**) Body weight gain, (**D**) daily calorie intake, and (**E**) energy efficiency ratio during the 6-week chronic light exposure and TRF intervention period. (**F**–**I**) Adipose tissue weight: (**F**) epididymal white adipose tissue (eWAT), (**G**) retroperitoneal white adipose tissue (retroWAT), (**H**) inguinal white adipose tissue (ingWAT), and (**I**) total adipose tissue (the sum of eWAT, retroWAT, and ingWAT). (**J**) eWAT weight and (**K**) liver mass are expressed as a function of body weight. (**L**) Inverse association between eWAT mass and liver weight in obese mice. (**M**) Liver weight. Lean-control: Mice fed a LFD ad libitum for 12 weeks under a control light condition (12 h light/dark cycle); Lean-24 h Light: Mice fed a LFD ad libitum for 12 weeks but exposed to chronic 24 h light for the last 6 weeks; Lean-24 h Light+TRF: Mice fed a LFD ad libitum for 6 weeks, then exposed to chronic 24 h light and a daily 10 h TRF for the last 6 weeks; Obese-control: Mice fed a HFD ad libitum for 12 weeks under a control light condition (12 h light/dark cycle); Obese-24 h Light: Mice fed a HFD ad libitum for 12 weeks but exposed to chronic 24 h light for the last 6 weeks; Obese-24 h Light+TRF: Mice fed a HFD ad libitum for 6 weeks, then exposed to chronic 24 h light and a daily 10 h TRF for the last 6 weeks. Data are presented as means ± SEM, n = 12 mice per group. * *p* < 0.05, ** *p* < 0.01, *** *p* < 0.001, **** *p* < 0.0001 by ANOVA with Tukey’s post hoc test.

Chronic light exposure has been shown to alter body weight or adiposity [3,17,18] and is often used to model human obesity associated with shift work [19]. To determine the effect of chronic light exposure alone or in combination with TRF on weight gain, we evaluated body weight, caloric intake, and energy efficiency ratio (EER) for both lean and obese mice under 12 h light (control), 24 h light, or 24 h light in combination with TRF (24 h light+TRF) (Figure 1A). During the light/TRF intervention period, the body weight of the obese-24 h light group was transiently higher than that of the obese-control group, but the obese-24 h light+TRF group had consistently lower body weight than both the obese-24 h light group and the obese-control group throughout the experiments (Figure 1B). In lean mice, body weight appeared to be comparable among groups (Figure 1B), but body weight gain relative to the initial body weight before intervention was significantly higher under chronic light exposure (5.91 ± 0.47 g vs. lean-control 4.48 ± 0.45 g), where TRF decreased it by ~40% (3.35 ± 0.39 g) (Figure 1C). Interestingly, chronic light exposure per se did not alter caloric intake in either lean or obese mice (Figure 1D), but the average caloric intake in the obese-24 h light group was significantly higher than that in the obese-24 h light+TRF group. When EER was used to compare the efficiency of animals in converting energy consumption into increased body weight, both lean and obese mice under chronic light exposure had higher EER values than their corresponding controls (Figure 1E). Furthermore, the TRF intervention not only eliminated the EER induction caused by the 24 h light conditions in both lean and obese mice, but also normalized the obesity-associated increase in EER, similar to that in the lean-control group. 

When looking at tissue weights (Figure 1F–M), an increase in both epididymal white adipose tissue (eWAT) (Figure 1F), retroperitoneal white adipose tissue (retroWAT) (Figure 1G) and, thus, total fat mass (Figure 1I) was observed in the lean-24 h light group. In line with several earlier studies [20,21,22], eWAT mass increased in mice with body weight up to ~40 g but eventually reduced with chronic obesity (Figure 1J), which was accompanied by a robust increase in liver mass (Figure 1K–M). While we did not detect a difference in fat and liver weight between the obese-control and obese-24 h light groups, the TRF intervention reduced obesity-associated eWAT loss (Figure 1F), inguinal white adipose tissue (ingWAT) expansion [20] (Figure 1H), and hepatomegaly (Figure 1M). These results indicate that chronic light exposure may increase susceptibility to weight gain per calorie consumed, while the TRF intervention may provide metabolic benefits beyond merely controlling dietary intake.

### 2.2. Effects of Chronic Light Exposure and TRF on Adipose Tissue Inflammation

**Figure 2:**
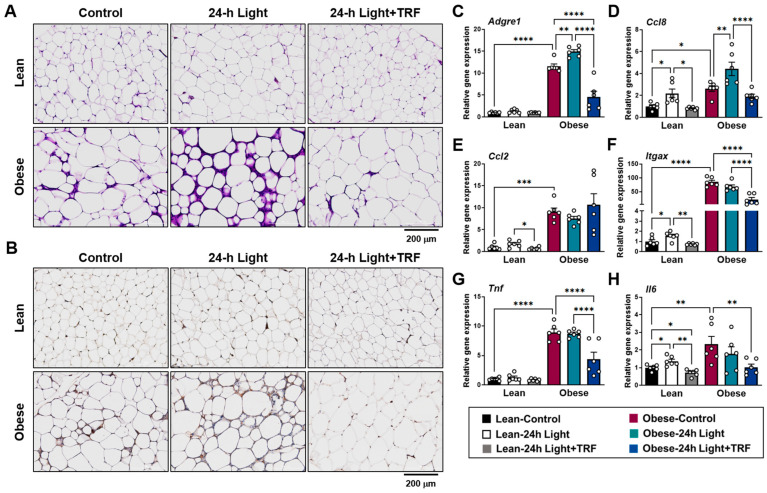
Effects of chronic light exposure and TRF on adipose tissue inflammation. (**A**,**B**) Representative images of epididymal adipose tissue stained with (**A**) H&E and (**B**) anti-F4/80 antibody. Scale bar = 200 μm. (**C**–**H**) Quantitative real-time PCR analysis of (**C**) macrophage-specific marker gene (*Adgre1*), (**D**) monocyte chemoattractant protein 2 (*Ccl8*), (**E**) monocyte chemoattractant protein 1 (*Ccl2*), (**F**) proinflammatory macrophage marker gene (*Itgax*), (**G**) proinflammatory cytokine (*Tnf*), and (**H**) proinflammatory cytokine (*Il6*) in epididymal fat. Data are presented as means ± SEM, n = 6 mice per group. * *p* < 0.05, ** *p* < 0.01, *** *p* < 0.001, **** *p* < 0.0001 by ANOVA with Tukey’s post hoc test.

Despite similar fat mass between the obese-control and obese-24 h light groups, H&E and F4/80 staining of eWAT showed significant infiltration of immune cells in the obese-24 h light mice but not in those under TRF intervention (Figure 2A), particularly F4/80-positive macrophages (Figure 2B). In line with this finding, gene expression analysis (Figure 2C–H) reveals an increase in the macrophage-specific marker gene *Adgre1* (F4/80) in the eWAT of the obese-24 h light group (Figure 2C). Consistent with this, the obese-24 h light group also exhibited higher expression of the monocyte chemoattractant *Ccl8* (Mcp2) (Figure 2D). Chronic light exposure also increased the expression of inflammatory macrophage marker genes, including *Itgax* (CD11c) (Figure 2F) and *Il6* (Figure 2H), as well as the monocyte chemoattractant *Ccl8* (Figure 2D), in lean mice. Most of these changes associated with chronic light exposure were normalized by TRF intervention in both lean and obese mice. Specifically, TRF intervention in obese mice subjected to 24 h light conditions significantly decreased *Adgre1* and *Itgax* levels by 60.5% and 74.4%, respectively, compared to those under control light conditions. Together, these results indicate that chronic light exposure may contribute to an increased proinflammatory response within adipose tissue, which can be greatly diminished by TRF intervention, especially in the obese state.

### 2.3. Effects of Chronic Light Exposure and TRF on Collagen Deposition and Fibrosis-Related Gene Expression in eWAT

**Figure 3:**
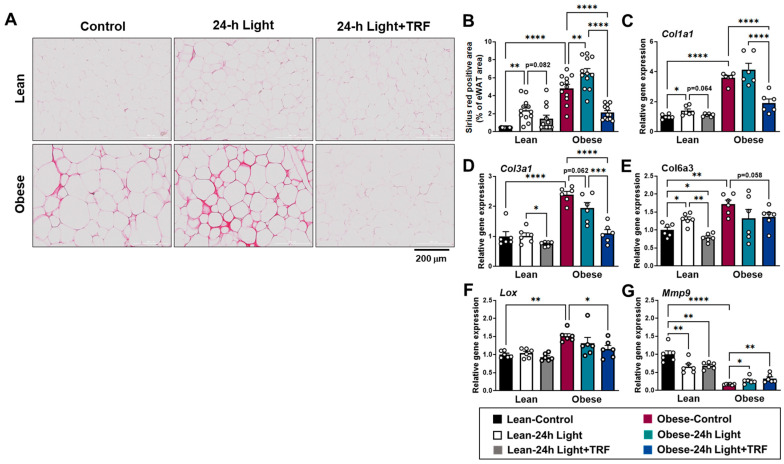
Effects of chronic light exposure and TRF on adipose tissue fibrosis. (**A**) Representative images of Picrosirius red-stained collagen in epididymal adipose tissues. Scale bar = 200 μm. (**B**) Picrosirius red-positive area. Data are presented as means ± SEM, n = 12 mice per group. (**C**–**G**) Quantitative real-time PCR analysis of (**C**) *Col1a1*, (**D**) *Col3a1*, (**E**) *Col6a3*, (**F**) *Lox*, and (**G**) *Mmp9* in epididymal fat. Data are presented as means ± SEM, n = 6 mice per group. * *p* < 0.05, ** *p* < 0.01, *** *p* < 0.001, **** *p* < 0.0001 by ANOVA with Tukey’s post hoc test.

In addition to WAT inflammation, fibrosis may also play a role in obesity complications via ectopic lipid accumulation in non-adipose tissue [23,24]. During the development of fibrosis, excess extracellular matrix (ECM) proteins are produced while their degradation is limited [25]. Collagens are the main components of the ECM in adipose tissue [26]. Histologically, Picrosirius red staining showed thicker and more collagen deposition in the interstitial space of the eWAT of obese mice, but not in those under TRF intervention (Figure 3A). Quantification of the stained area confirmed that the obese-control group had a significantly higher percentage of stained area in the eWAT compared to the lean-control group (Figure 3B). Moreover, chronic light exposure triggered more collagen deposition in both lean and obese mice. However, TRF intervention eliminated excess collagen deposition induced by obesity and chronic light exposure. 

Collagen types I, III, and VI are highly expressed in adipose tissue. Lysyl oxidase (LOX) is an enzyme responsible for collagen cross-linking, while matrix metalloproteinases are enzymes involved in the breakdown of ECM components, such as collagen. Consistent with the histological data, genes involved in collagen synthesis *Col1a1*, *Col3a1*, *Col6a3*, and *Lox* were upregulated in obese mice (Figure 3C–F), with a marked decrease in the gene involved in collagen breakdown, such as metalloproteinase *Mmp9*, to 16% of the levels observed in the lean-control mice (Figure 3G). In addition, chronic light exposure significantly increased *Col1a1* and *Col6a3* mRNA levels by up to 38% (Figure 3C,E) and decreased *Mmp9* mRNA level by 34% (Figure 3G) in the eWAT of lean mice. In contrast, obese mice exhibited a slight increase in *Mmp9* mRNA level when exposed to chronic light conditions, but still only at one-quarter of the level found in the lean-control group. Whether changes in fibrosis-related gene expressions were associated with obesity or chronic light exposure, TRF intervention effectively reduced these, except for the *Mmp9* gene. Taken together, TRF intervention protects against the development of adipose tissue fibrosis triggered by chronic light exposure alone in combination with obesity, possibly by controlling collagen synthesis rather than degradation.

### 2.4. Effects of Chronic Light Exposure and TRF on the Expression of Genes Involved in Lipid Metabolism in eWAT

**Figure 4:**
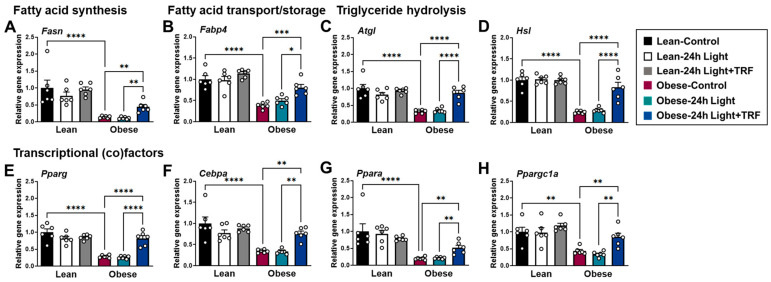
Effects of chronic light exposure and TRF on the expression of genes involved in lipid metabolism in epididymal adipose tissue. Quantitative real-time PCR analysis of (**A**) fatty acid synthase (*Fasn*), (**B**) fatty acid-binding protein 4 (*Fabp4*), (**C**) adipose triglyceride lipase (*Atgl*), (**D**) hormone-sensitive lipase (*Hsl*), and (**E**–**H**) transcriptional (co)factors in epididymal fat. Data are presented as means ± SEM, n = 6 mice per group. * *p* < 0.05, ** *p* < 0.01, *** *p* < 0.001, **** *p* < 0.0001 by ANOVA with Tukey’s post hoc test.

Since inflammation and fibrosis of adipose tissue are linked to its dysfunction [27,28], we investigated whether chronic light exposure and TRF could modulate the mRNA expression of genes involved in lipid metabolism in eWAT. The genes of interest include *Fasn*, which encodes fatty acid synthase, a key enzyme in the de novo synthesis of fatty acids used for the formation of triglyceride [29]. Fatty acid-binding protein 4 (FABP4), also known as aP2, binds to fatty acids and modulates lipid trafficking [30]. Conversely, lipolysis is primarily mediated by adipose triglyceride lipase (Atgl), followed by hormone-sensitive lipase (Hsl) [31]. As shown in Figure 4A–H, the expression of *Fasn*, *Fabp4*, *Atgl*, *Hsl*, and transcriptional (co)factor genes involved in lipid metabolism was substantially downregulated in the obese-control group compared to the lean-control group. While there was no difference between the control group and the 24 h light group, regardless of weight status, the TRF intervention reversed obesity-induced changes in the expression of genes involved in lipid metabolism in the eWAT.

### 2.5. Effects of Chronic Light Exposure and TRF on Hepatic Steatosis, Inflammation, and Fibrosis

**Figure 5:**
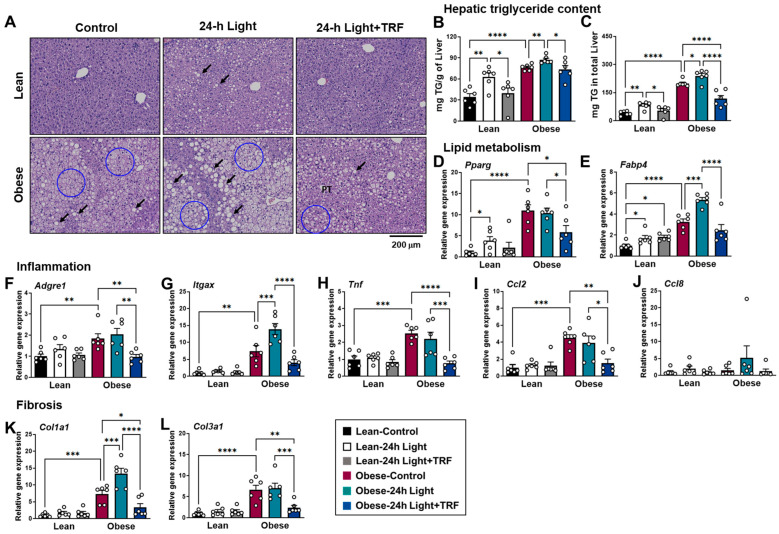
Effects of chronic light exposure and TRF on hepatic steatosis, inflammation, and fibrosis. (**A**) Representative H&E-stained histological sections of liver tissues. Macrovesicular lipid droplets (arrows) and microvesicular steatosis (circle) are present during the development of liver steatosis. Scale bar = 200 μm. (**B**,**C**) Hepatic triglyceride content adjusted by liver weight (**B**) or in total liver weight (**C**). (**D**–**L**) Quantitative real-time PCR analysis of (**D**) *Pparg*, (**E**) *Fabp4*, (**F**) *Adgre1*, (**G**) *Itgax*, (**H**) *Tnf*, (**I**) *Ccl2*, (**J**) *Ccl8*, (**K**) *Col1a1*, and (**L**) *Col3a1* in liver. Data are presented as means ± SEM, n = 6 mice per group. * *p* < 0.05, ** *p* < 0.01, *** *p* < 0.001, **** *p* < 0.0001 by ANOVA with Tukey’s post hoc test.

H&E staining of liver tissue showed lipid droplets, typically including both macrovesicular and microvesicular steatosis in obese groups, which were less obvious in those under TRF intervention (Figure 5A). Consistent with the histological data, hepatic triglyceride content was substantially increased in the obese-control group compared to the lean-control group (Figure 5B,C). In addition, chronic light exposure increased hepatic triglyceride content in both lean and obese mice, while TRF reduced it to levels observed in the absence of chronic light exposure (Figure 5B), with the effect being greater when accounting for differences in liver mass (Figure 5C). 

Gene expression analysis revealed an increase in lipogenic genes, such as peroxisome proliferator-activated receptor gamma (*Pparg*) and *Fabp4*, in the liver of the obese-control group, compared to the lean-control group (Figure 5D,E). Furthermore, the expression of the majority of genes involved in inflammation and fibrosis was upregulated during obesity (Figure 5F–L). In particular, the expression of the *Itgax* and *Col1a1* genes was further upregulated in obese mice by chronic light exposure. This upregulation was greatly decreased by TRF intervention, even to levels lower than those in the obese-control group. These results indicate that TRF intervention protects against hepatic triglyceride accumulation and the development of fatty liver, while also reducing the risk of liver inflammation and fibrosis during obesity combined with chronic light exposure.

## 3. Discussion

The global rise in obesity prevalence has coincided with increased exposure to light at night. While numerous studies suggest that TRF is associated with improvements in metabolic parameters related to obesity, it remains to be determined whether TRF is applicable to subjects exposed to light at night. In this study, we demonstrated that daily 10 h TRF interventions effectively mitigated weight gain caused by light exposure at night in both lean and obese mice, even when adjusted for lower caloric intake. Additionally, TRF normalized the majority of changes in gene expressions related to inflammatory and fibrotic markers in the eWAT and liver induced by light exposure at night, obesity, or both. Furthermore, light exposure at night induced hepatic triglyceride accumulation in both lean and obese mice, along with the upregulation of lipogenic genes (*Pparg*, *Fabp4*), which TRF reduced in the liver of obese mice. These results suggest that light exposure at night per se can act as an obesogenic, inflammatory, and fibrogenic factor in both lean and obese mice, all of which can be prevented by TRF intervention. 

How does TRF intervention reverse the detrimental effects of chronic light exposure? Our results show that 6 weeks of chronic light exposure did not alter calorie intake but induced more weight gain per calorie consumed in both lean and obese mice. Consistent with our observations, exposure to light at night has been demonstrated to increase body weight in mice despite comparable daily caloric intake [7,8]. This may be partly due to reduced total 24 h energy expenditure [6] and fat oxidation [32]. Our study reveals that TRF intervention lowered daily caloric consumption. However, the reduced efficiency of animals in converting consumption into body weight suggests that the anti-obesity effect of TRF cannot be solely attributed to changes in caloric intake. Similarly, TRF intervention was shown to restore the rhythmic oscillation of the respiratory exchange ratio (RER) in obese mice, with reduced RER during fasting, indicative of increased fat oxidation [11,33]. Thus, the restoration of daily feeding and fasting patterns through TRF intervention may provide benefits for weight management in conditions associated with chronic light exposure and/or shift work, beyond merely regulating dietary intake.

Additionally, TRF intervention reduced the infiltration of immune cells in eWAT during chronic light exposure. Adipose tissue macrophages (ATMs) are the predominant immune cells in obese adipose tissue and play a key role in adipose tissue inflammation. ATMs expressing CD11c are highly enriched during obesity, particularly in the crown-like structures surrounding dead or dying adipocytes [34]. Conversely, depletion of inflammatory CD11c^+^ macrophages in CD11c-DTR mice has been shown to reduce adipose tissue inflammation and obesity-induced insulin resistance [35]. It is important to note that, in our chronic light condition, mRNA expressions of the macrophage-specific marker gene *Adgre1*, particularly the proinflammatory macrophage markers *Itgax* (CD11c) and *Il6*, were upregulated in either lean or obese mice. Concomitantly, the mRNA expression of *Ccl8*, which is predominantly expressed by ATMs at much higher levels than *Ccl2* and mediates inflammatory monocyte recruitment [36], was greatly upregulated by chronic light exposure and downregulated by TRF intervention in mice, regardless of weight status. Previous studies have shown that increased chemokine expression in adipose tissue due to genetic modulation facilitates not only the infiltration of macrophages into adipose tissue but also hepatic steatosis [37]. We also observed that hepatic triglyceride content increased under chronic light exposure and decreased with TRF intervention, similar to changes in *Ccl8* gene expression. Thus, TRF intervention may protect against hepatic steatosis partly by modulating macrophage infiltration and activation in eWAT during obesity, chronic light exposure, or both. 

Excessive collagen accumulation and the development of fibrosis can limit adipocyte hypertrophy and lipid storage in adipose tissue, contributing to ectopic lipid accumulation and metabolic dysregulation [27,38]. While obese mice or individuals exhibit an upregulation of genes encoding collagen, such as types I, III, and VI [26,39,40], the deletion of collagen type VI can improve the metabolic phenotype of *ob*/*ob* mice fed a HFD [26]. Similarly, levels of degrading enzymes appear to be modulated in the obese state [38]. For instance, the mRNA expression of *Mmp9* was downregulated in the adipose tissue of C57BL/6 mice upon a 14-week HFD challenge [41]. In the current study, we found that chronic light exposure upregulated *Col1a1* and *Col6a3* transcripts while decreasing the *Mmp9* transcript in the eWAT of lean mice, along with increased collagen accumulation in both lean and obese mice. In addition, collagen genes induced by either chronic light exposure or obesity were normalized by TRF intervention, suggesting a role of TRF in counteracting adipose tissue fibrosis by regulating collagen synthesis. Interestingly, adipose tissue fibrosis can be attenuated by macrophage depletion [42], while overexpression of proinflammatory TNF-α promotes collagen accumulation around crown-like adipocytes [43]. Since inflammation during obesity is believed to be a key factor in adipose tissue fibrosis [44], we speculated that modulation of adipose tissue inflammation by TRF intervention at an earlier stage may, in turn, contribute to the prevention of fibrosis under chronic light exposure and obesity. 

Obesity is associated with the dysregulation of genes involved in lipid metabolism in eWAT. The downregulation of lipolytic and adipogenic gene expression in the eWAT of obese mice [21] was recapitulated in the current study. Although constant light exposure had minimal effects on adipogenic gene expression in both lean and obese mice, TRF intervention significantly mitigated the obesity-induced changes in adipogenic gene expression. *Pparg* overexpression in adipogenic precursors promotes healthy fat expansion characterized by fewer crown-like structures and reduced adipose tissue inflammation [45], suggesting that TRF provides metabolic benefits by normalizing adipogenic genes like *Pparg*. Concurrently, TRF intervention reversed the obesity-induced downregulation of peroxisome proliferator-activated receptor alpha (*Ppara*) and its coactivator *Ppargc1a*, which are crucial for activating genes involved in fatty acid catabolism. Despite *Ppara* being primarily expressed in metabolically active tissues such as the liver, its loss in adipocytes can lead to upregulation of iNOS, which mediates a shift toward inflammatory macrophages [46]. Additionally, TRF restored the mRNA expression of *Atgl* and *Hsl*, key enzymes in triglyceride breakdown [31], while a deficiency in the lipolytic enzyme Hsl in adipose tissue can cause hepatic steatosis [47]. Collectively, TRF-mediated normalization of lipid metabolism genes in eWAT may facilitate healthy adipose expansion with reduced inflammation and, consequently, less fat deposition in non-adipose tissues such as the liver during obesity. 

The present study also provides in vivo evidence that chronic light exposure induces hepatic triglyceride accumulation in both lean and obese mice. Hepatic triglyceride levels can be influenced by the supply of free fatty acids from lipolysis in adipose tissue, as well as by de novo triglyceride synthesis in the liver. While we did not observe differences in adipogenic and lipolytic gene expression in eWAT under chronic light exposure, there was a significant upregulation of the lipogenic gene *Pparg* and the fatty acid binding protein gene *Fabp4* in the liver. Although liver *Pparg* is not abundantly expressed under normal conditions, its upregulation has been observed in the livers of obese patients with steatosis and steatohepatitis [48]. Additionally, the mRNA expression of *Fabp4*, an intracellular lipid-binding protein most abundantly expressed in adipocytes and macrophages [49], was upregulated in the livers of morbidly obese patients with insulin resistance [50]. Not only can FABP4 induce intracellular lipid accumulation and cellular death in HepG2 liver cells [51], but it also enhances the inflammatory response in RAW264.7 macrophages [52]. We observed that the reduction in hepatic *Fabp4* expression through TRF intervention was associated with decreased hepatic triglyceride accumulation. Given that both genetic and pharmacological inhibition of FABP4 can alleviate inflammation and fibrosis [53,54], the TRF-mediated reduction in the mRNA expression of genes related to fibrotic markers (*Col1a1* and *Col3a1*) and the inflammatory marker (*Itgax*) suggests that FABP4 reduction may contribute to the therapeutic effects of TRF.

Cumulatively, we found that increased inflammation is a significant pathogenic event associated with chronic light exposure in both lean and obese mice. Additionally, there is evidence that long-term chronic light exposure induces oxidative stress in the rodent retina [55], brain [56], and liver [57]. Furthermore, bright light exposure before bedtime for three to four days resulted in circadian disruption in healthy individuals, accompanied by elevated oxidative stress markers [58]. Interestingly, deletion of the core circadian clock gene *BMAL1* in macrophages reduced the levels and activity of NRF2, a transcription factor that regulates the expression of antioxidant proteins [59]. Although BMAL1-deficient macrophages produced higher levels of reactive oxygen species and proinflammatory cytokines, their phenotype was rescued by pharmacological activation of NRF2 or antioxidant supplementation [59]. Thus, one potential mechanism underlying chronic light-induced inflammation may involve its association with increased oxidative stress. Whether antioxidant intervention, independently or synergistically with TRF, can offer protection under chronic light conditions or in shift workers warrants further investigation. 

We also acknowledge the limitations of our study, particularly the use of only young male mice. Young male mice are more prone to weight gain than female mice [60], which led us to select them to induce obesity within a shorter time frame and facilitate our research. However, menopause, characterized by age-related loss of ovarian function, is associated with weight gain and adipose tissue inflammation [60,61]. Additionally, older mice exhibit an increased ratio of fat to body weight, hepatic fat accumulation [62], and disrupted eating patterns when fed an obesogenic diet [63]. Notably, TRF blunted diet-induced weight gain and adipose tissue inflammation in both young and old male mice, but not in female mice [63]. However, TRF did provide protection against fatty liver in both male and female mice, regardless of age [63]. While we demonstrated the effects of TRF in young male mice, further studies are needed to understand how TRF-mediated protection is influenced by sex and age when challenged with chronic light exposure. Moreover, although our study employed 6 weeks of chronic light exposure in line with several rodent studies using similar interventions, those studies focused on different tissues of interest. A longer duration of chronic light exposure may amplify the differences in inflammatory and fibrotic gene expression in the eWAT of lean mice. However, eWAT mass eventually decreased with chronic obesity (Figure 1J), while there was a robust increase in liver mass (Figure 1K) [20,21,22]. This may explain the small differences in gene expression in eWAT between the obese-control and obese-24 h light groups. Therefore, varied time-course experiments may be necessary to further elucidate the pleiotropic effects of chronic light exposure on eWAT, liver, and other tissues. Nevertheless, our results imply that TRF reduced adipose tissue inflammation and fibrosis in young male mice under 6 weeks of chronic light exposure, regardless of weight status.

## 4. Materials and Methods

### 4.1. Animals and Experimental Design

All animal procedures were reviewed and approved by the Institutional Animal Care and Use Committee of Chungbuk National University (approval number: CBNUA-2079-23-01). Five-week-old C57BL/6J male mice were purchased from the Central Laboratory Animal Inc. (Seoul, Republic of Korea) and housed 4 mice per cage under controlled environmental conditions (23 °C ± 1 °C, 50% ± 10% humidity). All animals were entrained under standard light/dark 12 h:12 h conditions (lights on at 7 am) with ad libitum access to water and a low-fat diet (LFD; 10% kcal fat, D12450B; Research Diets, New Brunswick, NJ, USA). Following the 1-week adaptation period, all mice were randomly assigned to two groups and maintained on an LFD or switched to a HFD (60% kcal fat, D12492; Research Diets) for 6 weeks. At 12 weeks of age, mice fed either LFD or HFD were randomly divided into one of three experimental conditions for 6 weeks: (1) a 12 h light/12 h dark cycle with ad libitum access to diet (lean-control, obese-control), (2) exposure to 24 h constant light conditions (>100-lux light intensity) with ad libitum access to diet (lean-24 h light, obese-24 h light), and (3) exposure to 24 h constant light with a 10 h restricted feeding window (lean-24 h light+TRF, obese-24 h light+TRF), equivalent to the dark phase under standard light/dark conditions (ZT13-ZT23, one hour after lights off to one hour before lights on). Food access was controlled by transferring mice daily between cages with food and water, and cages with water only. Mice with ad libitum access to food were also transferred between feeding cages at the same time to control for mouse handling. 

### 4.2. Body Weight, Food Intake, and EER

Body weight was measured weekly and food intake was measured twice a week during the experiment. The EER was calculated as body weight gain divided by caloric consumption during the 6 weeks of light/TRF intervention, multiplied by 100.

### 4.3. Tissue Collection

At 16 weeks of age, mice were fasted with no food or feces in the bedding starting at 6 am. Mice were euthanized with isoflurane and tissues were harvested at 1 pm. Blood was collected in BD Microtainer serum separator tubes (365967, Becton Dickinson, Franklin Lakes, NJ, USA) via cardiac puncture. The liver and eWAT were weighed and placed in either 4% formaldehyde or liquid nitrogen for snap freezing and storage at −70 °C.

### 4.4. Histology and Immunohistochemistry

Left eWAT pads and liver tissues were fixed in 4% formaldehyde (Sigma-Aldrich, St. Louis, MO, USA) overnight, embedded in paraffin, sectioned, and stained with H&E. Macrophages in the paraffin-embedded eWAT were identified by immunohistochemical staining using a rat anti-F4/80 monoclonal antibody (1:100 dilution, sc-52664, Santa Cruz Biotechnology, Santa Cruz, CA, USA) and a Polink-2 Plus Rat HRP kit (OriGene, Rockville, MD, USA), with visualization achieved through diaminobenzidine staining (DAB; ScyTek Laboratories, Logan, UT, USA). Additionally, paraffin sections of fat tissues were stained with a Picrosirius red stain kit (ScyTek Laboratories) to visualize collagen and fibrosis in eWAT. Images were acquired using a BioTek Lionheart FX Cell Imager (Agilent, Santa Clara, CA, USA). To quantify the percentage area occupied by collagen fibers, at least three areas from each slide were photographed under 100× magnification and analyzed using ImageJ software (National Institutes of Health, Baltimore, MD, USA).

### 4.5. Hepatic Triglyceride

Liver tissues were snap-frozen in liquid nitrogen, and stored at −70 °C. Hepatic triglycerides were extracted and quantified using a triglyceride colorimetric assay kit (Ab65336, Abcam, Cambridge, UK). Briefly, lipids were extracted by homogenizing approximately 100 mg of liver tissue in 2 ml of 5% Igepal CA-630 (Sigma-Aldrich) in water, then heated at 90 °C in a water bath for 5 min. The samples were cooled down and then heated again to solubilize all triglycerides into solution. The samples were centrifuged for 5 min, and the supernatants were diluted 50-fold, combined with a triglyceride probe, enzyme mix, and lipase, and absorbance was measured at 570 nm using a SpectraMax iD3 plate reader (Molecular Devices, San Jose, CA, USA).

### 4.6. Quantitative Real-Time PCR

Right eWAT pads and liver tissues for quantitative real-time PCR (qPCR) measurements were excised, snap-frozen in liquid nitrogen, and stored at −70 °C until analysis. RNA was extracted from pulverized tissue using an RNeasy lipid mini kit (74804, Qiagen, Valencia, CA, USA) as indicated by the manufacturer. Total RNA (1 μg) was used for reverse transcription to cDNA using an Advantage RT for PCR kit (639506, Clontech, Palo Alto, CA, USA). qPCR was performed on a QuantStudio 5 Real-Time PCR system (Applied Biosystems, Foster City, CA, USA) using TaqMan Universal PCR Master Mix (4304437, Applied Biosystems) and primers for *Adgre1* (Mm00802529_m1), *Atgl* (Mm00662319_m1), *Ccl2* (Mm00441242_m1), *Ccl8* (Mm01297183_m1), *Cebpa* (Mm00514283_s1), *Col1a1* (Mm00801666_g1), *Col3a1* (Mm00802300_m1), *Col6a3* (Mm00711678_m1), *Fabp4* (Mm00445878_m1), *Fasn* (Mm00662319_m1), *Hsl* (Mm00495359_m1), *Il6* (Mm00446190_m1), *Itgax* (Mm00498698_m1), *Lox* (Mm00495386_m1), *Mmp9* (Mm00442991_m1), *Ppara* (Mm00440939_m1), *Pparg* (Mm00440940_m1), *Ppargc1a* (Mm01208835_m1), *Tbp* (Mm00446973_m1), and *Tnf* (Mm00443258_m1) (all FAM probes, Applied Biosystems) as follows: one cycle at 95 °C (10 min), 40 cycles of 95 °C (15 s) and 60 °C (1 min). Fold changes were calculated as 2^−ΔΔCt^ compared with the endogenous control gene, TATA box binding protein (TBP), using the lean-control group as the reference.

### 4.7. Statistical Analysis

Results are expressed as means ± SEM. Data were analyzed using ANOVA followed by Tukey’s HSD post hoc procedure. Pearson correlation test was conducted to evaluate the association between variables. All statistical analyses were carried out using Prism version 9 software (GraphPad, San Diego, CA, USA) or SPSS version 25.0 software (SPSS Inc., Chicago, IL, USA), with *p* values smaller than 0.05 considered significant.

## 5. Conclusions

In summary, we demonstrate that chronic light exposure increases susceptibility to weight gain per calorie consumed, regardless of weight status. Additionally, chronic light exposure serves as a pathogenic factor in the onset and progression of adipose tissue inflammation and fibrosis, as well as in the development of fatty liver. Whether due to chronic light exposure alone or in combination with obesity, our studies provide compelling evidence for the preventive and therapeutic role of TRF in managing adipose tissue inflammation and hepatic steatosis. These results suggest that individuals exposed to light at night may benefit from TRF intervention; however, clinical studies are needed to confirm these findings.

## Data Availability

The data presented in this study are available on request from the corresponding author.
